# Serotonin's many meanings elude simple theories

**DOI:** 10.7554/eLife.07390

**Published:** 2015-04-08

**Authors:** Peter Dayan, Quentin Huys

**Affiliations:** Gatsby Computational Neuroscience Unit, University College London, London, United Kingdomdayan@gatsby.ucl.ac.uk; Translational Neuromodeling Unit, Institute for Biomedical Engineering, University of Zurich & ETH Zurich, Zurich, Switzerland and Department of Psychiatry, Psychotherapy and Psychosomatics, University of Zurich, Zurich, Switzerlandqhuys@cantab.net

**Keywords:** neurophysiology, reward, punishment, behavior, serotonin, dorsal raphe, mouse

## Abstract

Neurons that produce serotonin respond in a number of different and complex ways in anticipation and receipt of rewards or punishments.

**Related research article** Cohen JY, Amoroso MW, Uchida N. 2015. Serotonergic neurons signal reward and punishment on multiple timescales. *eLife* **4**:e06346. doi: 10.7554/eLife.06346

**Image** Serotonin (cyan) and channelrhodopsin (magenta) labelled in the midbrain of a mouse

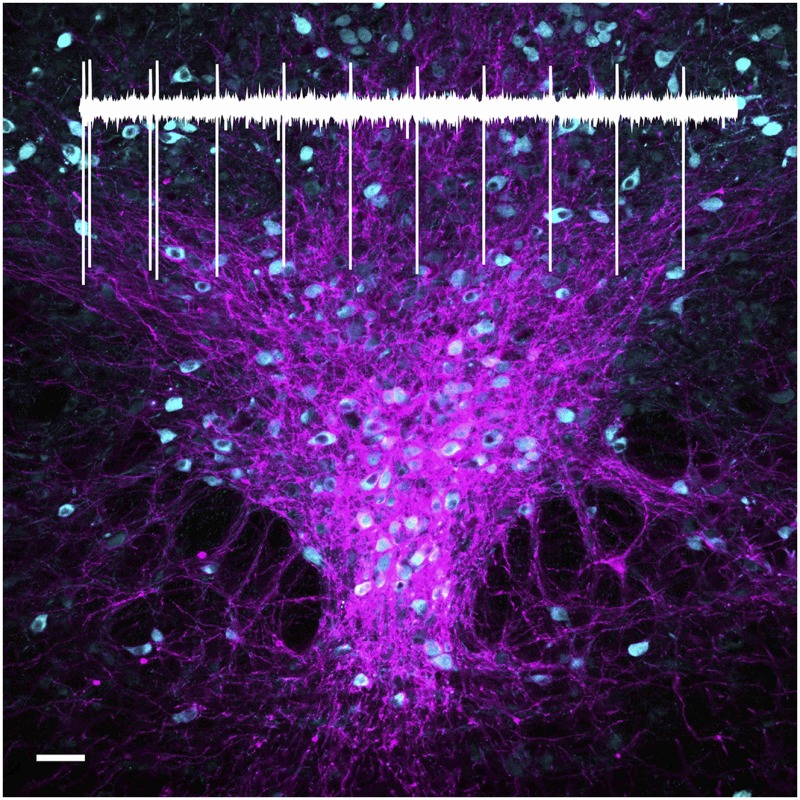

Dopamine and serotonin are neuromodulators. Produced by small assemblies (or nuclei) of neurons deep in the brain stem, these molecules are projected throughout the brain to regulate the excitability and plasticity of broad neural networks via a fiendishly complex cast of receptor types. The importance of neuromodulators is underscored by their involvement in a wealth of neurological and psychiatric diseases. What has been harder to pin down are the details of their computational roles, particularly the semantics of what they signal. Now, in *eLife*, Jeremiah Cohen, Mackenzie Amoroso and Naoshige Uchida add much-needed data about the activity of neurons that release serotonin in a task involving predictable rewards and punishments (Cohen et al., 2015). These data nicely muddy the theoretical waters.

The past two decades have ascribed dopamine a particularly crisp computational role. Seminal electrophysiological recordings suggested that the phasic activity of dopamine-producing neurons—the brief spikes in electrical activity seen after a stimulus is applied—closely resembles a sophisticated form of ‘prediction error’ that can be used to learn how much reward to expect and then influence the choice of appropriate actions. Interpreting electrophysiological recordings, however, has always been difficult.

Neuromodulatory neurons reside in complex nuclei that harbour many different types of neurons, raising doubts about whether any recorded electrophysiological activity can really be related to particular neuromodulators. Such doubts have largely been settled for dopamine by Cohen, Uchida and co-workers at Harvard University (Cohen et al., 2012) using optogenetic tagging: this technique allows the dopamine neurons to be electrophysiologically identified by genetically modifying them so that they can be stimulated with light (Lima et al., 2009).

Serotonin, by comparison, has been more elusive. There is a rather broad, though not completely self-consistent, cluster of electrophysiological, pharmacological, depletion- and lesion-based results suggesting that serotonin might play a critical role in preventing active behaviours or deciding to withdraw from a situation. In this role, it is often associated with the anticipation and/or delivery of a punishment (Deakin and Graeff, 1991; Schweimer et al., 2008; Amo et al., 2014). More recent optogenetic evidence that serotonin is involved in patience could be at least partially related to this (Miyazaki et al., 2014). Along with more direct findings, these results have collectively, if somewhat controversially, been discussed in terms of serotonin (putatively linked with punishment and inhibition) and dopamine (putatively linked with reward and activation) playing opposing roles (Deakin and Graeff, 1991).

However, there is both electrophysiological and optogenetic evidence that serotonin is involved in many other roles, such as rhythmic motor activity (Ranade and Mainen, 2009). There is also recent, direct, evidence for its association with reward (Liu et al., 2014). Indeed, the fact that selective serotonin reuptake inhibitors (SSRIs) are the major treatment for depression has always hinted at a role for serotonin in the ascription or use of positive values. The mooted explanation for serotonin's role in this process—that the positive associations arise from adaptions that produce appropriate responses to losses (Dayan and Huys, 2008)—seems unlikely to suffice in the face of all this contrary evidence.

Here, Cohen (who is now at Johns Hopkins University), Amoroso and Uchida (who are both at Harvard University) used optogenetic tagging to identify the serotonergic neurons of mice in a brain area called the dorsal raphe nucleus (Cohen et al., 2015). They then studied the activation of these cells in awake animals under a Pavlovian conditioning paradigm. In blocks of trials, particular odours preceded a reward (water), a punishment (bitter-tasting quinine, or an airpuff to the face) or nothing, so that the mice learned to associate an odour with a particular outcome. The first, sobering, finding was that both tagged and untagged neurons show a substantial diversity in their electrical activity and the aspects of the behaviour with which this activity was correlated. This shows the likely impossibility of classifying whether a neuron is serotonergic without some form of molecular proof.

In addition, the results add substantially to our knowledge about the complex relationship between the activity of serotonergic neurons and rewards and punishments. There are three key responses to consider: the baseline activity just before each odour, potentially reflecting the level of reward or punishment of the block; the activity inspired by the odour; and the activity produced by the outcome that the odour predicts.

Very crudely, blocks of rewards elicited greater tonic activity—that is, more sustained firing—between trials in serotonin neurons than blocks of punishments (although the opposite pattern was also apparent). Such a link of tonic activity to the average level of reward had previously been proposed for dopamine rather than serotonin (Niv et al., 2007). Strikingly, when Cohen and colleagues recorded from dopamine neurons they failed to find such a signal. How tonic serotonin represents average reward is, however, complicated: though responding more to rewards than losses, serotonin neurons mostly decreased their tonic firing rates as the size of the average reward increased. Nevertheless, the phasic responses of the neurons to reward-predicting odours were more prominent than those to punishment-predicting odours. Conversely, the actual delivery of a punishment produced more pronounced phasic activity than the delivery of a reward. This latter finding is consistent with a class of neurons recorded in anaesthetized animals (Schweimer et al., 2008).

This notable paper by Cohen and colleagues is credibly the end of the end of theories of serotonin acting as an aversive counterpart to dopamine. It may also be the end of the beginning of a new wave of results (Schweimer et al., 2008; Amo et al., 2014; Liu et al., 2014; Miyazaki et al., 2014) that have reinforced a richly varied picture of this neuromodulator's role in motivation and emotion. The beginning of the end of our befuddlement might come through using markers or methods that allow neurons activated during behaviour to be re-activated experimentally (such as the conditional expression of channelrhodopsin in activated serotonergic neurons). This could allow the motley collection of neural subgroups observed in the dorsal and median raphe nuclei (Lowry et al., 2005) to be further resolved.
